# Dark Current Analysis on GeSn *p-i-n* Photodetectors

**DOI:** 10.3390/s23177531

**Published:** 2023-08-30

**Authors:** Soumava Ghosh, Greg Sun, Timothy A. Morgan, Gregory T. Forcherio, Hung-Hsiang Cheng, Guo-En Chang

**Affiliations:** 1 Department of Mechanical Engineering, and Advanced Institute of Manufacturing with High-Tech Innovations (AIM-HI), National Chung Cheng University, Chiayi 621301, Taiwan; ghoshsoumava2@gmail.com; 2 Department of Engineering, University of Massachusetts—Boston, Boston, MA 02125, USA; greg.sun@umb.edu; 3 Electro-Optic Technology Division, Naval Surface Warfare Center, Crane, IN 47522, USA; timothy.a.morgan45.civ@us.navy.mil (T.A.M.); gregory.t.forcherio.civ@us.navy.mil (G.T.F.); 4 Center for Condensed Matter Sciences and Graduate Institute of Electronics Engineering, National Taiwan University, Taipei 106, Taiwan; hhcheng@ntu.edu.tw

**Keywords:** GeSn alloys, defects, dark current, detectivity, sustainability

## Abstract

Group IV alloys of GeSn have been extensively investigated as a competing material alternative in shortwave-to-mid-infrared photodetectors (PDs). The relatively large defect densities present in GeSn alloys are the major challenge in developing practical devices, owing to the low-temperature growth and lattice mismatch with Si or Ge substrates. In this paper, we comprehensively analyze the impact of defects on the performance of GeSn *p-i-n* homojunction PDs. We first present our theoretical models to calculate various contributing components of the dark current, including minority carrier diffusion in *p*- and *n*-regions, carrier generation–recombination in the active intrinsic region, and the tunneling effect. We then analyze the effect of defect density in the GeSn active region on carrier mobilities, scattering times, and the dark current. A higher defect density increases the dark current, resulting in a reduction in the detectivity of GeSn *p-i-n* PDs. In addition, at low Sn concentrations, defect-related dark current density is dominant, while the generation dark current becomes dominant at a higher Sn content. These results point to the importance of minimizing defect densities in the GeSn material growth and device processing, particularly for higher Sn compositions necessary to expand the cutoff wavelength to mid- and long-wave infrared regime. Moreover, a comparative study indicates that further improvement of the material quality and optimization of device structure reduces the dark current and thereby increases the detectivity. This study provides more realistic expectations and guidelines for evaluating GeSn *p-i-n* PDs as a competitor to the III-V- and II-VI-based infrared PDs currently on the commercial market.

## 1. Introduction

Infrared (IR) photodetectors (PDs) are important optical sensors for a wide range of applications, such as fiber-optic communications, Lidar, gas-sensing, and thermal version. The III-V- and II-VI based photodetectors have gained strong traction on the IR commercial market due to their high detectivity in shortwave-infrared (SWIR) (*λ* = 1–3 µm) and mid-infrared (MIR) regions (*λ* = 3–5 µm) at room temperature (*T* = 300 K), as well as 77 K [1,2,3,4,5,6,7,8,9,10,11,12]. However, their incompatibility with the Si-based complementary metal–oxide–semiconductor (CMOS) processing technology makes them expensive and complex to integrate with Si readout circuits, thus restricting their application scope. On the other hand, the group IV semiconductors, i.e., Si- and Ge-based devices, have attracted attention because of their CMOS compatibility, monolithic integrability on the same Si or silicon-on-insulator (SOI) chips, and lower fabrication cost with respect to the III–V- and II–VI-based devices. However, the limited cutoff wavelengths ~1.1 µm and 1.5 µm of the Si-and Ge-based PDs, respectively, make them unsuitable for the recent telecommunication window (1.55 µm), as well as MIR applications [13,14,15].

The successful development of both strained and unstrained high-quality group-IV Ge_1–x_Sn_x_ alloys on Si or SOI substrates via a suitable buffer layer, using either low-temperature chemical vapor deposition (CVD) or molecular beam epitaxy (MBE) growth techniques, has changed the prospect dramatically in last two decades [16,17,18]. The incorporation of Sn, another group-IV element, into Ge reduces the direct bandgap faster than the indirect bandgap of the alloy, leading to direct-bandgap GeSn alloys when the Sn concentrations reach beyond a certain critical value (~6–8%) [19]. In addition, Ge_1–x_Sn_x_ alloys also provide a wide range of bandgap tunability, a large absorption coefficient [19], a high carrier mobility [20], and a high carrier saturation velocity [21]. These noteworthy features have made Ge_1–x_Sn_x_ alloys a promising material for PDs that can cover the entire SWIR range and a good portion of the MIR region up to 3.7 μm [21,22,23,24,25,26,27,28,29]. Furthermore, the presence of the L-valley in the conduction band enables a special momentum-space carrier separation scheme in favor of high-performance photodetection [30]. Recently, a theoretical analysis of the defect-free GeSn *p-i-n* PDs in the MIR region indicated that their achievable performance can indeed compete with the existing III-V- and II–VI-based PDs, showing great promise for eventual low-cost MIR photodetection [19]. However, the reported GeSn-based PDs to date usually suffer from relatively high levels of defect densities owing to the low-temperature growth and lattice mismatch with the Si or Ge substrates. Despite the continuous advances in material growth, defects are not likely to disappear in GeSn alloys in the near future, significantly impacting the dark current, which is one of the most critical parameters of IR PDs. Although there have been numerous reports about the dark current densities of GeSn-based PDs [31,32,33,34,35,36,37,38,39,40], very little has been done to clarify the various contributors to the dark-current-density GeSn PDs with different Sn concentrations and the effect of defect density, limiting the optimization of GeSn PDs to achieve uncooled and high-performance MIR photodetection. A few recent experimental studies have shed light on the contributing components of dark currents, such as minority carrier diffusion, Shockley–Read–Hall (SRH) generation–recombination (GR), and trap-assisted tunneling (TAT), providing evidence that the dark current of GeSn PDs is lower with lower defect densities [41,42,43]. This intended to further study the effect of defect density on the PD performance by establishing a theoretical model upon which material and device developers can form realistic expectations in developing GeSn PD architectures for various applications.

In this work, for the first time, we present a comprehensive theoretical study of device performance of GeSn PDs in terms of the Sn concentrations and defect density. Starting from analyzing the impact of defect densities on the carriers’ mobility and scattering time, we examine different components contributing to the dark current, including minority carrier diffusion, carrier generation–recombination, and tunneling. We then calculate the responsivity and detectivity and discuss the dependences of the defect density and Sn composition on detectivity.

The rest of the paper is organized as follows: the envisioned structure of the GeSn *p-i-n* PD is described in Section 2; the analytical modeling of the mobility and scattering time under different defect densities are discussed in Section 3; the theoretical model of the dark current is presented in Section 4; the performance analysis on such envisioned architecture is discussed in Section 5 and Section 6; and, finally, the conclusion is offered in Section 7.

## 2. Device Structure of GeSn *p-i-n* PDs

**Figure 1:**
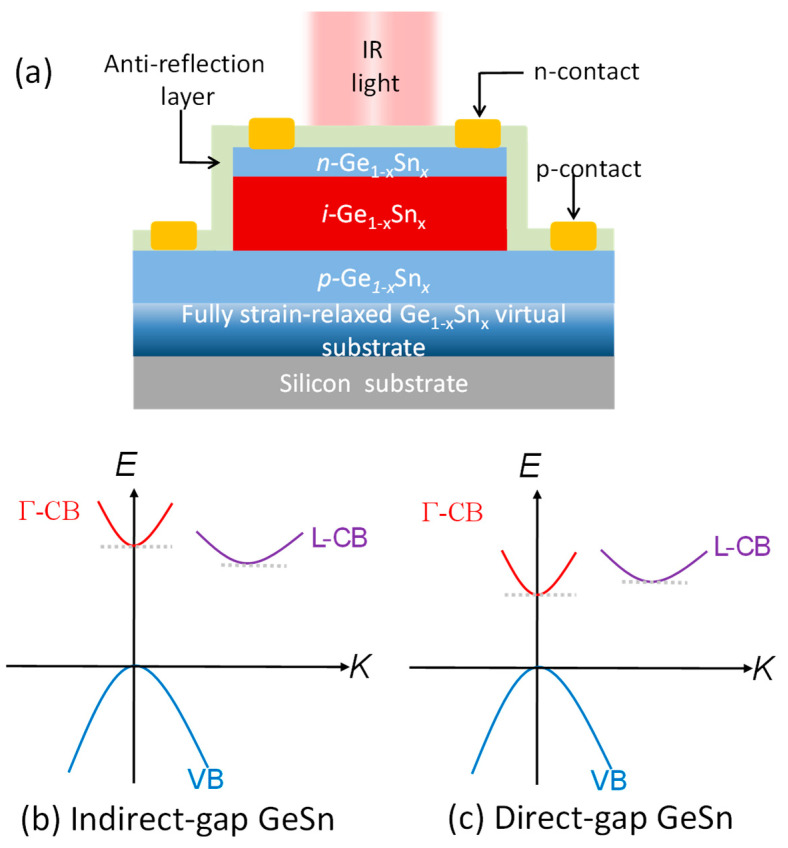
(**a**) Schematic diagram of the lattice-matched, strain-free Ge_1–x_Sn_x_ *p-i-n* PD on a Si (001) substrate via a fully strain-relaxed, lattice-matched, compositionallygraded Ge_1–x_Sn_x_ buffer layer (not to scale). Schematic band structures of (**b**) indirect-bandgap GeSn and (**c**) direct-bandgap GeSn layers.

The conceptual structure of the homojunction Ge_1–x_Sn_x_ *p-i-n* PD is shown in Figure 1a. A composition-graded fullystrainrelaxed Ge_1–x_Sn_x_ buffer layer is first grown on a Si (001) substrate. Then, a lattice-matched strain-free Ge_1–x_Sn_x_ *p-i-n* structure is grown, where an intrinsic Ge_1–x_Sn_x_ layer with a thickness of *t*_i_ = 3000 nm is sandwiched between a heavily doped (*N*_a_ = *N*_d_ = 1 × 10^19^ cm^−3^) *n*-type Ge_1–x_Sn_x_ layer with a thickness of *t*_n_ = 100 nm and a *p*-type Ge_1–x_Sn_x_ layer with a thickness of *t*_p_ = 500 nm. The compositional fraction, *x*, is presumed to be the same in all layers. Therefore, we can neglect the effect of the strain introduced by the latticemismatch with the increase in the Sn concentration. Similar GeSn *p-i-n* PD structures on silicon substrates via a GeSn buffer have been experimentally demonstrated [42]. An anti-reflection coating (ARC) is deposited on top of the PD to minimize the reflection of the incident light in order to increase the light absorption. This ARC also serves as the passivation layer to minimize the surface dark current [9,10], which is not considered in this study. The defect density in the Ge_1–x_Sn_x_ *p-i-n* PD is assumed to be *N*_def_. With different Sn concentrations, the Ge_1–x_Sn_x_ can be an indirect-bandgap (Figure 1b) and a direct-bandgap material (Figure 1c). Here, we consider the GeSn PD to operate at zero bias conditions, so the dark current density is minimal, and the detectivity is maximal. Using the junction theory, the built-in electric field across the *i*-GeSn region is estimated to be *F*~6 kV/cm.

The energy bandgaps of Ge_1–x_Sn_x_alloys that determine the photodetection range of GeSn PDs can be calculated as follows [19]:

(1) $$E_{g}^{\xi }\left({\mathrm{Ge}}_{1-x}{\mathrm{Sn}}_{x}\right)=\left(1-x\right)\times E_{g}^{\xi }\left(\mathrm{Ge}\right)+x\times E_{g}^{\xi }\left(\mathrm{Sn}\right)-x\times \left(1-x\right)\times b_{\xi }$$

where ξ denotes either the Γ- or L-valley in CB; $E_{g}^{\xi }\left(\mathrm{Ge}\right)$ and $E_{g}^{\xi }\left(\mathrm{Sn}\right)$ represent the bandgap energies of Ge and Sn, respectively; and $b_{\xi }$ is the bowing parameters. The values used in this calculation aretaken from Reference [19].

**Figure 2:**
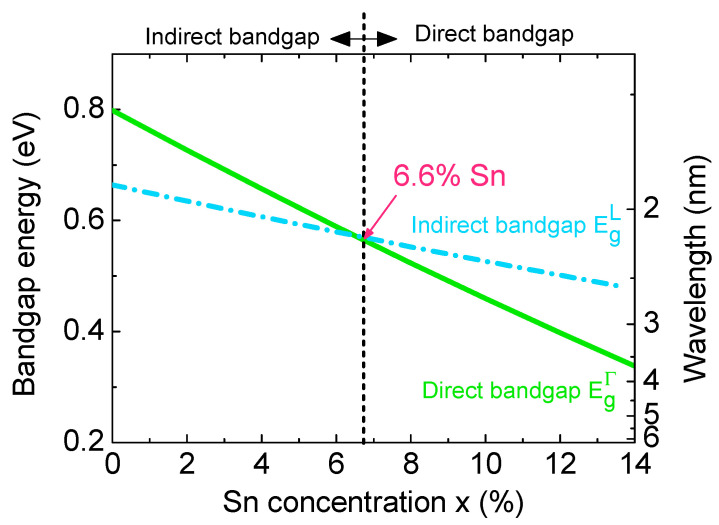
Calculated indirect and direct bandgaps of bulk Ge_1–x_Sn_x_ as a function of the Sn concentration at *T* = 300 K.

Figure 2 shows the variation in the indirect and direct bandgaps as a function of the Sn concentration at room temperature (*T* = 300 K).The increase in the Sn concentration decreases the indirect and direct bandgaps of GeSn, that remarkably redshifts both the indirect- and direct-band absorption edges. Thus, efficient MIR detection can be achieved in GeSn PDs. Due to the negative direct bandgap of Sn, the direct bandgap of Ge_1–x_Sn_x_ decreases at a faster rate than the indirect bandgap and finally crosses it at ~6.6%. Therefore, beyond ~6.6% Sn concentration, Ge_1–x_Sn_x_ is transformed to a direct bandgap material.

## 3. Mobilities and Scattering Times

In this section, we formulate and calculate the carriers’ mobility and scattering time of Ge_1–x_Sn_x_ alloys in the presence of defect density.

Dislocation densities in the intrinsic region have been considered to be the predominant defect centers that obstruct a carriers’ movement, resulting in a reduction in carrier mobilityand scattering time. We consider three different mobilities: electrons in the Γ-valley conduction band (Γ-CB) and L-valley conduction band (L-CB) and holes in the valence band (VB). In the presence of defects, they can all be expressed as follows [44,45]:

(2) $$\mu_{e}=\frac{30\sqrt{2\pi }\varepsilon^{2}d^{2}{\left(k_{B}T\right)}^{3/2}}{N_{\mathrm{def}}q^{3}f_{\mathrm{def}}^{2}L_{d}\sqrt{m_{ce}^{*}}}$$

(3) $$\mu_{h}=\frac{30\sqrt{2\pi }\varepsilon^{2}d^{2}{\left(k_{B}T\right)}^{3/2}}{N_{\mathrm{def}}q^{3}{\left(1-f_{\mathrm{def}}\right)}^{2}L_{d}\sqrt{m_{ch}^{*}}}$$

where *ε* is permittivity of the Ge_1–x_Sn_x_; *q* is the electronic charge; *k_B_* is the Boltzmann constant; *T* is the temperature; *d* is the distance between the defect centers (can be estimated from the defect density via $N_{\mathrm{def}}\left({\mathrm{cm}}^{-2}\right)=10^{14}/\left(\pi d^{2}\right)$ [46]); *f*_def_ is the Fermi occupation factor of ionized defect centers (approximated as ~0.5, assuming they are situated near the center of the bandgap of the intrinsic region); and $m_{ce}^{*}$ and $m_{ch}^{*}$ denote the conductivity effective masses of electron and hole, respectively, that depends on the Sn composition of alloys taken from the 30-band full-zone *k*·*p* model [47]. The Debye screening length is $L_{d}\left[={\left(\varepsilon k_{B}T/q^{2}n_{i}\right)}^{1/2}\right]$ [45], where *n_i_* is the intrinsic carrier concentration that depends on the GeSn alloy bandgap governed by the Sn concentration.

**Figure 3:**
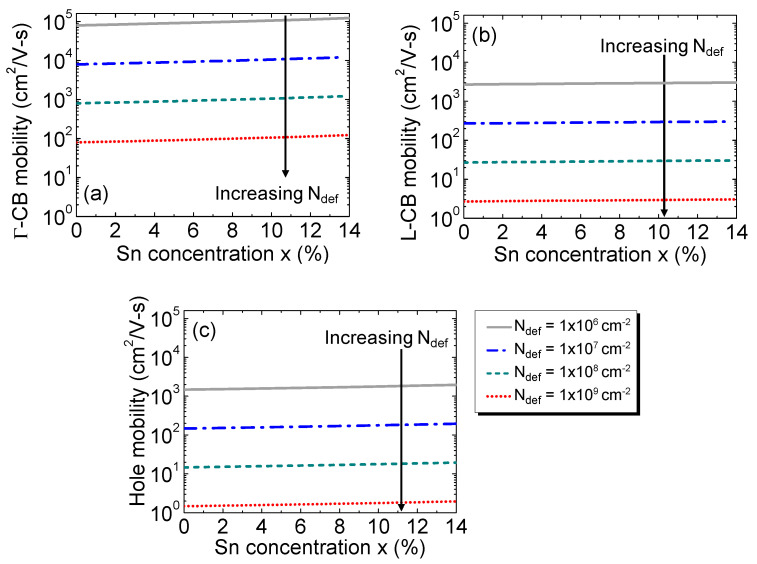
Mobility of the electrons in the (**a**) Γ-CB, (**b**) L-CB, and (**c**) holes as a function of the Sn concentration for different defect densities.

The calculated electron mobilities at the Γ-CB and L-CB and the hole mobility as a function of the Sn concentration with various defect densities are shown in Figure 3. The results show that the mobilities significantly decrease with the increasing defect density. Concomitantly, mobility increases modestly with the increasing Sn concentration because of the reduction in the conductivity mass $m_{ce}^{*}$, and the Debye length as a result of the increased intrinsic carrier concentration.

The carrier scattering time is related to the mobility as follows [45]:

(4) $$\tau =\frac{m_{c}^{*}\mu }{q}$$

**Figure 4:**
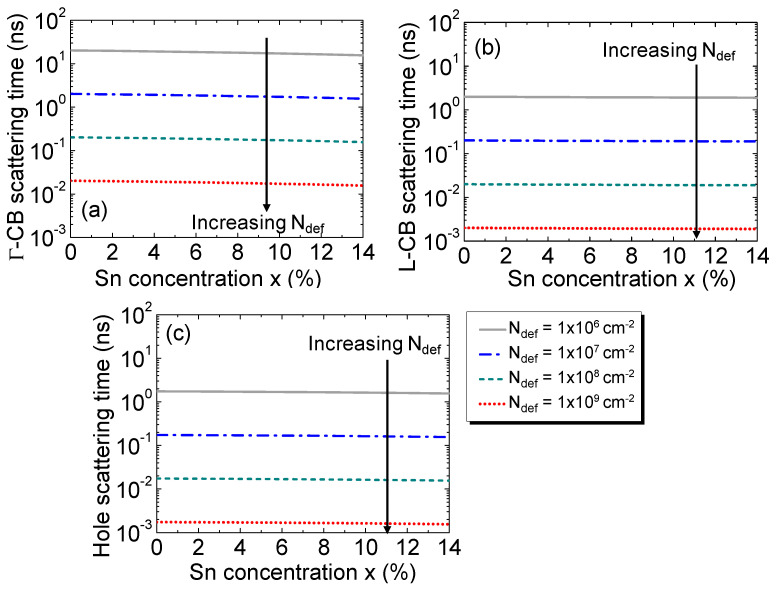
Scattering times of the electrons in (**a**) Γ-CB, (**b**) L-CB, and (**c**) holes as a function of the Sn concentration for various defect densities.

Their dependences on the Sn concentration are shown for Γ-CB and L-CB electrons and holes in Figure 4a–c, respectively, for several defect densities. In addition to the rapid reduction in the scattering time as the defect density increases, the increase in the Sn concentration, on the other hand, results in a small decrease in the scattering time because of the reductions in the conductivity mass and the Debye length that tends to compensate for each other at a fixed defect density.

## 4. Dark Current Analysis

Dark current density is an important parameter of the PD, andseveral mechanisms contribute to the dark current density, including the diffusion of the minority carriers towards the intrinsic region from the heavily doped contact layers (*J*_diff_), the GR current due to the presence of defect states acting as the GR centers inside the intrinsic region, carrier tunneling, and the surface leakage current [9,10].The GR current density consists of (a) SRH (*J*_SRH_) or trap-assisted, (b) interband generation (*J*_gen_), and (c) Auger or three-carrier processes (*J*_Aug_). The tunneling component generally occurs due to the tunneling of the carriers and is of two types: TAT (*J*_TAT_) and band-to-band tunneling (BTBT). The thicker intrinsic region (~3000 nm) in the GeSn PDs under consideration prevents the BTBT. Furthermore, owing to the lattice-matched strain-relaxed structure, the defect density is negligible for the surface. As a result, the surface leakage current [48] is not considered. Therefore, the reverse saturation current density (*J*_0_) can be expressed as the summation of these contributing mechanisms, as shown below:

(5) $$J_{0}=J_{\mathrm{diff}}+J_{\mathrm{SRH}}+J_{\mathrm{gen}}+J_{\mathrm{Aug}}+J_{\mathrm{TAT}}$$

Next, we show our theoretical models for different dark current components and present the calculation results. The material parameters of GeSn alloys are obtained from the linear interpolation of those of Ge and Sn taken from Reference [49].

### 4.1. Diffusion Dark Current Density

**Figure 5:**
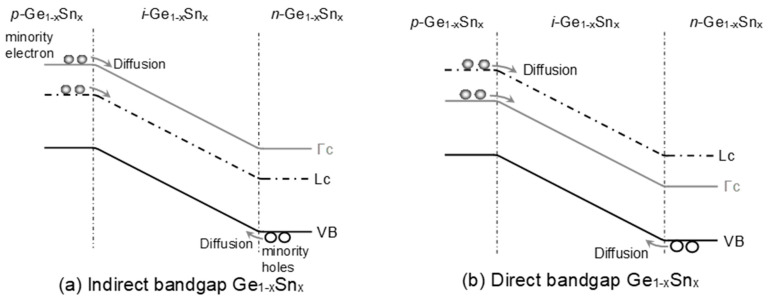
Illustration of dark current due to diffusion of minority carriers in homojunction GeSn PD with (**a**) indirect-bandgap and (**b**) direct-bandgap GeSn layers.

Figure 5 depicts the generation of a diffusion dark current due to the diffusion of minority holes (electrons in Γ-CB and L-CB) towards the *i*-Ge_1–x_Sn_x_ layer from the heavily doped *n*-Ge_1–x_Sn_x_ (*p*-Ge_1–x_Sn_x_) layer. 

The minority carriers’ diffusion current density under the short-base approximation can be calculated as follows [19]:

(6) $$J_{\mathrm{diff}}=q\left[\frac{D_{n}^{\Gamma }}{t_{p}}n_{p0}^{\Gamma }+\frac{D_{n}^{L}}{t_{p}}n_{p0}^{L}+\frac{D_{p}}{t_{n}}p_{n0}\right]$$

where $D_{n}^{L}$, $D_{n}^{\Gamma }$, and $D_{p}$ are the diffusion coefficients of electrons in the L-CB, Γ-CB, and holes, respectively, which are related to the mobility via $D=\mu k_{B}T/q$, and obviously they are also a function of defect density; and *t*_p_ and *t*_n_ denote the thickness of the heavily doped *p*- and *n*-type Ge_1–x_Sn_x_ layer. The minority concentrations are related to the doping concentrations and the intrinsic carrier concentration (*n_i_*) by $(n_{p0}^{\Gamma }+n_{p0}^{\Gamma })=n_{i}^{2}/N_{a}$ and $p_{n0}=n_{i}^{2}/N_{d}$.The intrinsic carrier concentration in the GeSn alloy can be expressed as the sum of contributions from the Γ- ($n_{i}^{\Gamma }$) and L-CB ($n_{i}^{L}$), as expressed below [19]:

(7) $$\begin{array}{l} n_{i}^{2}={\left(n_{i}^{\Gamma }+n_{i}^{L}\right)}^{2}\\ =N_{C}^{\Gamma }N_{V}\exp\left(-\frac{E_{g}^{\Gamma }}{k_{B}T}\right)+N_{C}^{L}N_{V}\exp\left(-\frac{E_{g}^{L}}{k_{B}T}\right) \end{array}$$

(8a) NCΓ=2(2πmΓ*kBTℏ2)32

(8b) NCL=2(2πmL*kBTℏ2)32

(8c) NV=2(2πmh*kBTℏ2)32

where *ħ* is the reduced Planck’s constant; $E_{g}^{\Gamma }$ and $E_{g}^{L}$ represent the direct- and indirect-bandgap energies of the Ge_1–x_Sn_x_ alloy, respectively, taken from Reference [19]; $N_{C}^{\Gamma }$, $N_{C}^{L}$, and *N_V_* are the effective density-of-state (DOS) values for electrons in the Γ-CB, L-CB, and holes in the VB, respectively [19]; and $m_{\Gamma }^{*}$, $m_{L}^{*}$, and $m_{h}^{*}$ are the DOS effective masses of electrons in the Γ-CB, L-CB, and holes, respectively, with their values taken from the 30-band full-zone *k*·*p* model [47]. The lifetime of electrons in Γ- and L-valley are considered as 12 ns and 100 μs [19] respectively while the hole lifetime $\tau_{p}$ = 100 μs [19]. 

**Figure 6:**
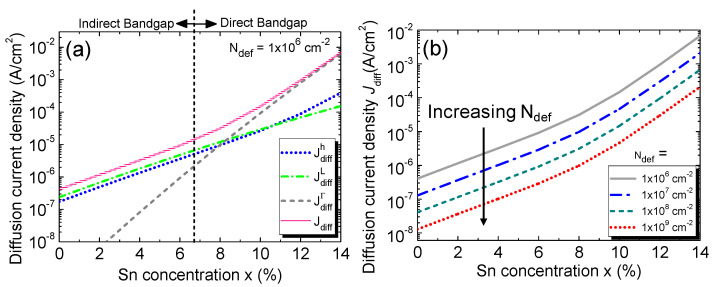
(**a**) Minority carrier diffusion current density and its different contributing components as a function of the Sn concentration with a defect density of 10^6^ cm^−2^. (**b**) Minority carrier diffusion current density as a function of the Sn concentration with various defect densities.

The diffusion dark current density, along with its various contributing components, is shown in Figure 6a for *N*_def_ = 1 × 10^6^ cm^−2^. The increase in the Sn concentration not only shrinks the bandgap of the alloy but also increases the intrinsic carrier concentration [19]. As a result, the minority carrier diffusion into the *i*-GeSn region increases and thereby increases the diffusion current density. For lower Sn concentrations, the L-CB sits below the Γ-CB, so the GeSn is still an indirect bandgap material. Thus, more electrons reside in the L-CB than in the Γ-CB in the *p*-doped region, so the L-CB component is more dominant. On the other hand, beyond the indirect-to-direct transition, the Γ-CB becomes lower than the L-CB, and the Γ-CB component increases sharply and ultimately crosses the L-CB component after reaching a Sn composition of ~8% and becomes dominating. The hole component is always lower than that of electrons because of its lower mobility.

Next, we show the calculated diffusion current density as a function of the Sn concentration with various defect densities in Figure 6b. At a fixed defect density, the diffusion current increases with the increasing Sn concentration, owing to the increased intrinsic carrier density. As the defect density increases, the carriers’ scattering time, mobility, and diffusion coefficients decrease, resulting in a reduction in the minority carrier diffusion current density.

### 4.2. SRH Dark Current Density

**Figure 7:**
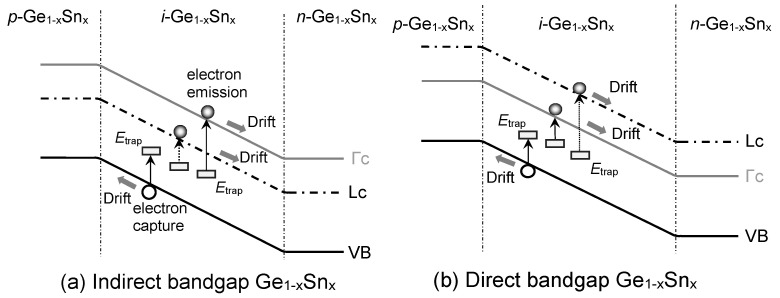
Illustration of SRH dark currents in GeSn PD with (**a**) indirect-bandgap and (**b**) direct-bandgap GeSn layers.

The dark current density induced by either capturing an electron from the VB while leaving a hole or emitting an electron to the CB by the defect states (*E*_trap_) situated in bandgap is an SRH or trap-assisted current. Figure 7 illustrates the SRH current inside the *i*-Ge_1–x_Sn_x_ layer for both direct- and indirect-bandgap Ge_1–x_Sn_x_PDs in which contributions from both the Γ-CB and L-CB, as well as the VB, are accounted for by the following [50]:

(9) $$J_{\mathrm{SRH}}=J_{\mathrm{SRH}}^{\Gamma }+J_{\mathrm{SRH}}^{L}+J_{\mathrm{SRH}}^{h}=q\left[A_{e}^{\Gamma }n_{i}^{\Gamma }+A_{e}^{L}n_{i}^{L}+A_{h}n_{i}\right]\times t_{i}$$

where the SRH coefficients of electrons in the Γ-CB ($A_{e}^{\Gamma }$) and L-CB ($A_{e}^{L}$) and the holes in the VB ($A_{h}$) are as follows [50]:

(10a) $$A_{e}^{\Gamma }=\sigma_{e}v_{\mathrm{th}}^{\Gamma }N_{\mathrm{def}}N_{\mathrm{TD}}$$

(10b) $$A_{e}^{L}=\sigma_{e}v_{\mathrm{th}}^{L}N_{\mathrm{def}}N_{\mathrm{TD}}$$

(10c) $$A_{h}=\sigma_{h}v_{\mathrm{th}}^{h}N_{\mathrm{def}}N_{\mathrm{TD}}$$

where *σ*_e_ (*σ*_h_) is the electron (hole) capture cross-sectional area, $v_{\mathrm{th}}=\sqrt{3k_{B}T/m_{c}^{*}}$ is the thermal velocity of the carriers, and *N*_TD_is the number of traps per unit length of defect. The capture cross-sectional area of the electron at room temperature is approximated to that of pure Ge (*σ*_e_ = 7 × 10^–16^ cm^2^ [51]), owing to the lack of experimental data on GeSn alloys. For holes, we considered the value of the capture cross-sectional area of the GeSn alloy from Gupta et al.’s work to be *σ*_h_ = 1.5 × 10^–19^ cm^2^ [52].

**Figure 8:**
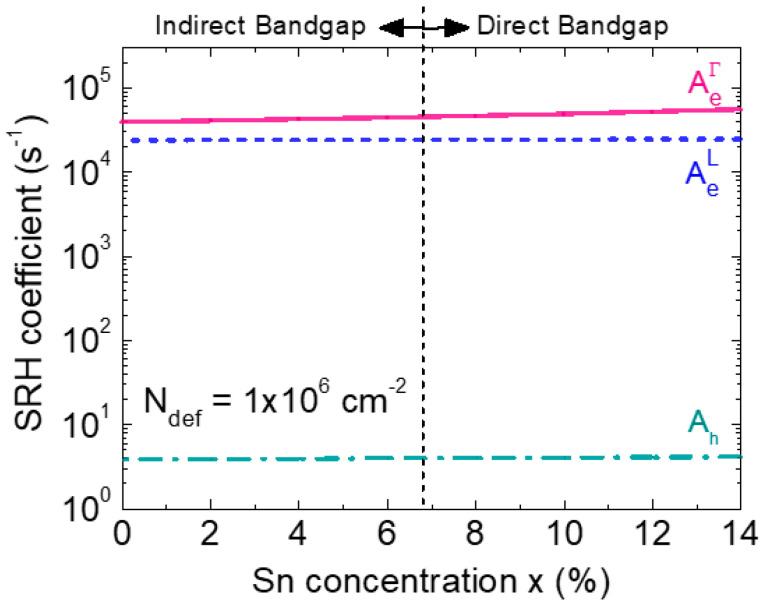
SRH coefficients for electrons and holes of GeSn alloys as a function of the Sn concentration.

Figure 8 shows the calculated SRH coefficients as a function of the Sn concentration for *N*_def_ = 1 × 10^6^ cm^−2^. Because the lowest effective mass of electron belongs to the Γ-CB, its thermal velocity is much higher than that of the electron in the L-CB and hole. Thus, the SRH recombination coefficient of the electron in Γ-CB is the highest among them. The thermal velocity increases with the increase in the Sn concentration due to the reduction in the effective masses. Therefore, the SRH recombination coefficient shows an increasing trend with the increase in the Sn concentration.

**Figure 9:**
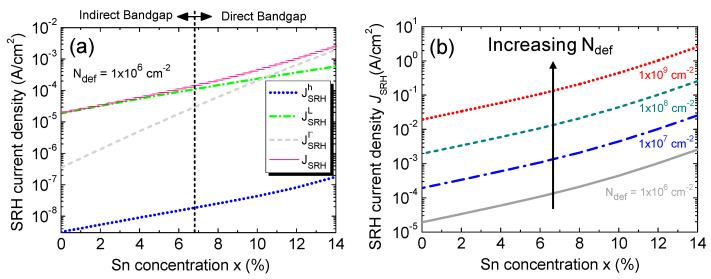
(**a**) SRH current density, along with its contributing components, as a function of the Sn concentration at a fixed defect density of 1 × 10^6^ cm^−2^. (**b**) SRH current density as a function of the Sn concentration for various defect densities.

Figure 9a depicts the calculated SRH current density against the Sn concentration with a fixed defect density of *N*_def_ = 1 × 10^6^ cm^−2^. The increase in the Sn concentration reduces both the direct and indirect bandgap of the GeSn alloy and increases the intrinsic carrier concentration [19]. As a result, more electron–hole pairs (EHPs) are generated due to the capture and emission of electrons by the defect states present inside the intrinsic Ge_1–x_Sn_x_ region at higher Sn concentrations. Thus, the SRH current density increases with the increasing Sn concentration. With low Sn concentrations, more electrons emitted by the defect states land in L-CB, producing a larger L-CB component than that of Γ-CB. As Ge_1–x_Sn_x_ becomes a direct-bandgap material at higher Sn concentrations, the Γ-CB component increases rapidly and eventually surpasses the L-CB component when the Sn concentration is higher than ~10%. Since electrons have a higher thermal velocity than holes, the electron SRH current density of either Γ-CB or L-CB is always higher than that of the hole one. Figure 9b shows the SRH current density as a function of the Sn concentration for various defect densities. The increase in the defect density increases the GR rate of the carriers by the defect states. Thus, the SRH current density increases rapidly with the increasing defect density.

### 4.3. Generation Dark Current Density

**Figure 10:**
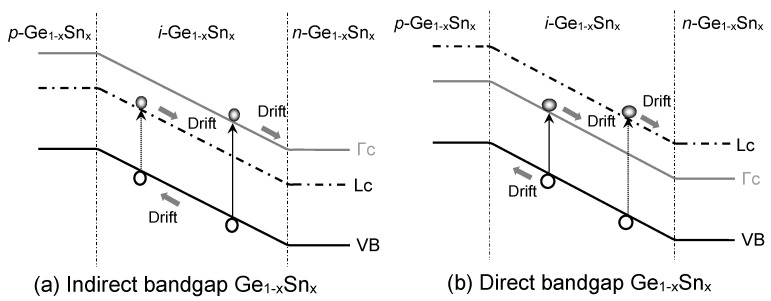
Illustration of dark current due to the generation of EHPs in homojunction GeSn PD with (**a**) indirect-bandgap and (**b**) direct-bandgap Ge_1–x_Sn_x_ layers.

Figure 10 illuminates the mechanisms of the generation dark current in GeSn PDs, where the band-to-band interband transitions in the *i*-Ge_1–x_Sn_x_ region also contribute to the dark current. EHPs are created thermally in the intrinsic Ge_1–x_Sn_x_ layer and then swept by the built-in electric field to form dark currents.

To calculate the generation dark current in the GeSn’s active region, we start with the calculation of the absorption coefficient of the Ge_1–x_Sn_x_ alloy. In Ge_1–x_Sn_x_, the photon absorption can take place via direct-gap transition (VB→Γ-CB) and indirect-gap (VB→L-CB) interband transitions. The absorption coefficient (α_dir_) for direct transition can be calculated using Fermi’s golden rule, considering the Lorentzian line-shape function and the nonparabolicity effect as follows [53]:

(11) $$\alpha_{\mathrm{dir}}\left(E\right)=\frac{\pi \hbar q^{2}}{n_{r}c\varepsilon_{0}m_{0}^{2}E}\sum_{m}\int \frac{2dk}{{\left(2\pi \right)}^{3}}{\left|\hat{q}.p_{CV}\right|}^{2}\times \frac{\gamma /2\pi }{{\left[E_{C\Gamma }\left(k\right)-E_{m}\left(k\right)-E\right]}^{2}+{\left(\gamma /2\right)}^{2}}$$

where *n_r_* represents the refractive index of the active medium; *c* is the velocity of light in vacuum; *ɛ*_0_ is the free space permittivity; *m*_0_ is the rest mass of electron; *ω* denotes the angular frequency of incident light; *E* is the incident photon energy; ${\left|\hat{q}.p_{CV}\right|}^{2}=m_{0}E_{P}/6$ indicates the momentum matrix, with $E_{p}$ denoting the optical energy parameter; *γ* is the full-width-at-half-maximum (FWHM) of the Lorentzian line-shape function; and *E_CΓ_*(**k**) and *E_m_*(**k**) denote the electron and hole energy in the Γ-CB and VB, respectively, which can be calculated using a multi-band *k*·*p* method [53], and the summation is over all interband transitions, from VB (both heavy hole and light hole) to Γ-CB.

The indirect absorption coefficient (α_indir_), on the other hand, can be calculated by considering acoustic phonon absorption and emission processes, using the following empirical expression [21]:

(12) $$\alpha_{\mathrm{indir}}\left(E\right)=A_{p}{\left(E-E_{g}^{L}+E_{ap}\right)}^{2}+A_{p}{\left(E-E_{g}^{L}-E_{ap}\right)}^{2}$$

where the first term is associated with acoustic phonon absorption, $\left(E>E_{g}^{L}-E_{ap}\right)$, and the second term is associated with acoustic phonon emission, $\left(E>E_{g}^{L}+E_{ap}\right)$, and E_ap_ is the energy of acoustic phonon. Experimental data on *A_p_* and *E_ap_* are not currently available for Ge_1–x_Sn_x_. Because of the similarity of the band structures between Ge_1–x_Sn_x_ and Ge, the values for Ge_1–x_Sn_x_ are approximated to those of Ge (*A_p_* = 2717 cm^–1^ and *E_ap_* = 27.7 meV at room temperature [21]). The total optical absorption coefficient (α) can then be calculated as follows:

(13) $$\alpha \left(E\right)=\alpha_{\mathrm{dir}}\left(E\right)+\alpha_{\mathrm{indir}}\left(E\right)$$

**Figure 11:**
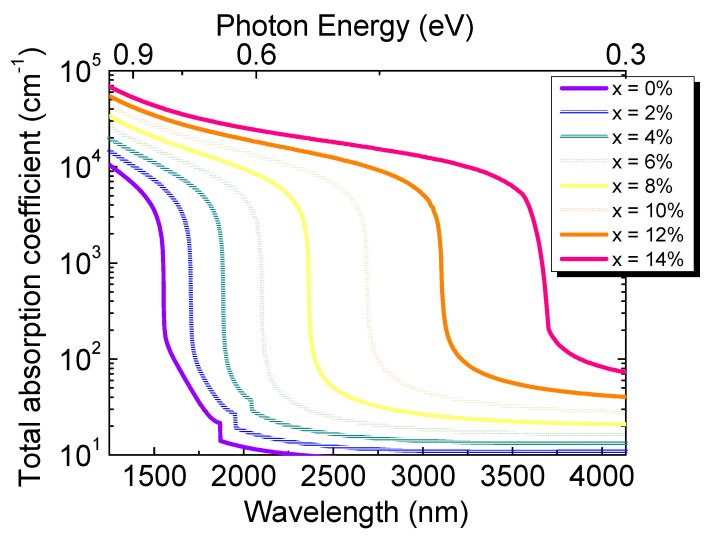
Calculated total absorption coefficient spectra of GeSn alloys with different Sn concentrations.

The calculated result of total optical absorption coefficient spectra is shown in Figure 11 for a range of Sn concentrations. It can be noted that, for a particular Sn concentration, the total absorption coefficient decreases with the increase in the wavelength, followed by a sharp decrease near the direct bandgap energy. With the increase in the Sn concentration, the bandgap energy decreases, causing the redshift of the cutoff wavelength of Ge_1–x_Sn_x_ PDs.

The direct-gap ($R_{0}^{\Gamma }$) and indirect-gap absorption rate per unit volume ($R_{0}^{L}$) can be calculated according to van Roosbroeck–Schockley model, as follows [54,55]:

(14) $$R_{0}^{\Gamma }\left(E\right)=\int_{0}^{\infty }\frac{n_{r}^{2}}{\pi^{2}\hbar^{3}c^{2}}\frac{\alpha_{\mathrm{dir}}\left(E\right)E^{2}}{\exp\left[\left(E-q\varphi \right)/k_{B}T\right]-1}dE$$

(15) $$R_{0}^{L}\left(E\right)=\int_{0}^{\infty }\frac{n_{r}^{2}}{\pi^{2}\hbar^{3}c^{2}}\frac{\alpha_{\mathrm{indir}}\left(E\right)E^{2}}{\exp\left[\left(E-q\varphi \right)/k_{B}T\right]-1}dE$$

where *qϕ* denotes the energy difference between the quasi-fermi levels of the*n*- and *p*-GeSn regions. Under equilibrium condition, the direct-gap ($B_{eh}^{\Gamma }$) and indirect-gap bimolecular recombination coefficients ($B_{eh}^{L}$) can be calculated as follows [54]:

(16) $$B_{eh}^{\Gamma }=\frac{R_{0}^{\Gamma }\left(E\right)}{N_{C}^{\Gamma }N_{V}\exp\left(-\frac{E_{g}^{\Gamma }}{k_{B}T}\right)}$$

(17) $$B_{eh}^{L}=\frac{R_{0}^{L}\left(E\right)}{N_{C}^{L}N_{V}\exp\left(-\frac{E_{g}^{L}}{k_{B}T}\right)}$$

The generation current density in Ge_1–x_Sn_x_can then be calculated as follows [53]:

(18) $$J_{\mathrm{gen}}=J_{\mathrm{gen}}^{\Gamma }+J_{\mathrm{gen}}^{L}=q\left[B_{eh}^{\Gamma }n_{i}^{\Gamma }n_{i}+B_{eh}^{L}n_{i}^{L}n_{i}\right]\times t_{i}$$

**Figure 12:**
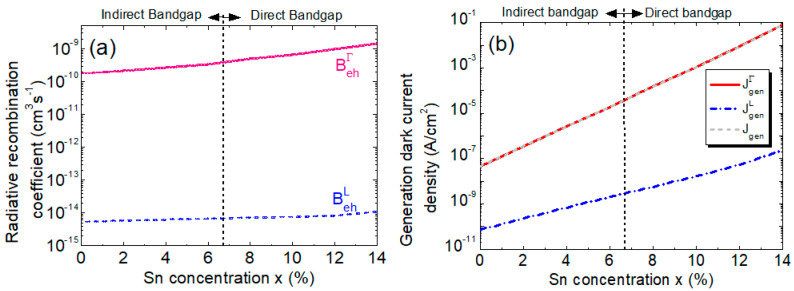
(**a**) Radiative recombination coefficients as a function of the Sn concentration. (**b**) Different contributors and total interband generation dark current density as a function of the Sn concentration.

To validate the calculation results, we first compare our calculation for Ge_1–x_Sn_x_ at x = 0 (i.e., pure Ge) with the reported experimental data for Ge [56], as shown in Table 1. A good agreement is found. Figure 12a depicts the calculated direct and indirect radiative recombination coefficients calculated as a function of the Sn concentration. As the Sn concentration increases, so does the absorption coefficient, which, in turn, leads to the increase in the recombination coefficients in the infrared region. Because the Γ-CB bandgap drops down faster than the L-CB with the increasing Sn concentration, the Γ-valley radiative recombination coefficient increases more rapidly than the L-valley one.

Figure 12b depicts the calculated generation dark current density, along with its contributing components. The increase in the Sn concentration causes the bandgaps to decrease and the intrinsic carrier concentration to increase, which, in turn, leads to an increase in both the direct and indirect generation rates. As a result, the total generation dark current density increases significantly with the increase in the Sn concentration. In addition, it is not difficult to see from Figure 12b that the direct-gap generation dark current dominates over its indirect counterpart because L-CB electrons require phonon participation to induce interband transitions, which are far less efficient processes than that of Γ-CB. The difference becomes more pronounced with higher Sn concentrations, particularly when Ge_1–x_Sn_x_ becomes direct-gap materials.

### 4.4. Auger Dark Current Density

**Figure 13:**
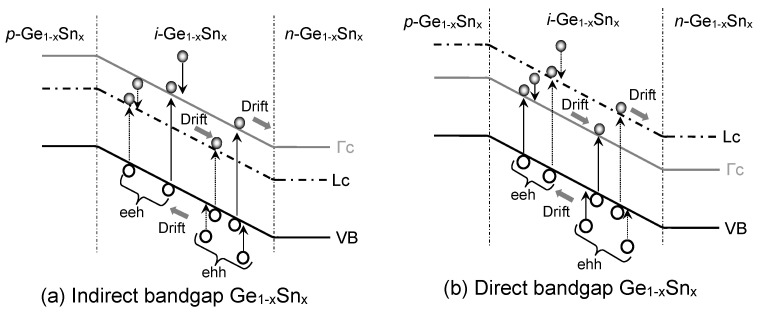
Illustration of Auger dark current due to eeh and ehh processes in (**a**) indirect-bandgap and (**b**) direct-bandgap Ge_1–x_Sn_x_ alloys.

Auger or three-carrier GR is another important contributor to the dark current, especially when the carrier concentrations are high. Such a process takes place when an electron or hole relaxes from a higher energy level to a lower energy level while transferring its energy to create an EHP, as illustrated in Figure 13. Those generated EHPs can then produce an Auger dark current. Depending on the participating carriers, the Auger process can be designated as either electron–electron–hole (eeh) or electron–hole–hole (ehh). Needless to say, the Auger current is significant only at higher carrier concentrations. Here, we shall illustrate the calculation of an eeh Auger coefficient. The ehh Auger coefficient can be calculated in a similar way. The Auger coefficient involving the eeh process can be calculated as follows [57]:

(19) $$C_{eeh}=\frac{m_{e}^{*}\alpha_{c}^{2}{\left|M_{a}\right|}^{2}}{4\pi^{4}\hbar^{3}{\left(1+2m_{r}\right)}^{3/2}}\frac{I_{0}}{N_{C}^{2}N_{V}}\exp\left(-\frac{E_{T}-E_{g}}{k_{B}T}\right)$$

(20) $$m_{r}=m_{e}^{*}/m_{h}^{*}$$

(21) $$\alpha_{c}=\left(m_{h}^{*}+m_{e}^{*}\right)/\left(m_{h}^{*}+2m_{e}^{*}\right)$$

where $m_{e}^{*}$ is the DOS effective mass of electrons in CB, $E_{T}\left(=E_{g}/\alpha_{c}\right)$ is the threshold energy required for the second electron to participate in the Auger process, ${\left|M_{a}\right|}^{2}$ is the matrix elements of coulomb interaction between two electrons at threshold energy with $k_{T}=\sqrt{\left(2m_{e}^{*}/\hbar^{2}\right)E_{T}}$, and *I*_0_ is expressed as [57]

(22) $$I_{0}=\int_{0}^{\infty }k^{2}{\left(k+k_{T}\right)}^{2}{\left(k+2k_{T}\right)}^{2}\times \exp\left[-\frac{\hbar^{2}}{2m_{e}^{*}k_{B}T}k\left(k+2k_{T}\right)\right]dk$$

Because of the close proximity between Γ-CB and L-CB, both contribute to the Auger current density, which can be calculated as follows:

(23) $$J_{\mathrm{Aug}}=J_{\mathrm{Aug}}^{\Gamma }+J_{\mathrm{Aug}}^{L}+J_{\mathrm{Aug}}^{h}=q\left[C_{\mathrm{eeh}}^{\Gamma }{\left(n_{i}^{\Gamma }\right)}^{2}n_{i}+C_{eeh}^{L}{\left(n_{i}^{L}\right)}^{2}n_{i}+C_{ehh}n_{i}^{3}\right]\times t_{i}$$

**Figure 14:**
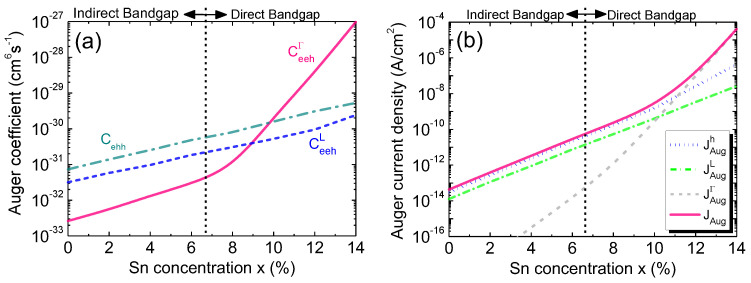
(**a**) Auger coefficients as a function of the Sn concentration. (**b**) Auger current density and its contributing components as a function of the Sn concentration.

To validate our model, we first calculate the Auger coefficients for pure Ge (*x* = 0%) and compare our results with the experiment data in the literature [56]. The comparison is shown in Table 2, where excellent agreement is found. Next, we calculate the Auger coefficients as a function of the Sn concentration for eeh and ehh processes involving either L-CB or Γ-CB electrons, and the results are shown in Figure 14a. From Equation (19), it can be observed that the Auger coefficients are dependent on bandgap energy, as well as the effective DOS in the energy bands. Auger coefficients increase with the increasing Sn concentration because of the reduction in both the bandgap and effective DOS. Moreover, the direct-bandgap Ge_1–x_Sn_x_ at a higher Sn concentration produces a larger Auger coefficient than that of the indirect-bandgap Ge_1–x_Sn_x_, thus dominating the Auger process. Figure 14b shows our calculated Auger current density, along with its contributing components, as a function of the Sn concentration. A higher Sn concentration leads to a higher intrinsic carrier concentration [19], thereby producing a larger Auger current. At a lower Sn concentration, when Ge_1–x_Sn_x_ is indirect, most electrons reside in the L-CB. As a result, the L-CB Auger dark current dominates over the Γ-CB component. However, as the Sn concentration increases, more and more electrons reside in the Γ-CB, and the trend becomes reversed, so the Γ-CB Auger process eventually surpasses both the L-CB and ehh Auger components and becomes dominant.

### 4.5. Tunneling Dark Current Density

**Figure 15:**
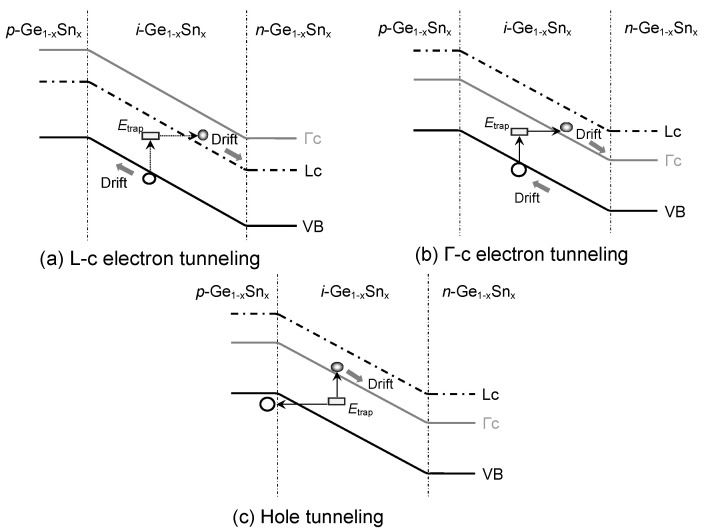
Illustration of TAT-related electron tunneling in Ge_1–x_Sn_x_PD with (**a**) indirect-bandgap and (**b**) direct-bandgap GeSn alloys and (**c**) hole tunneling.

Figure 15 illustrates the mechanisms of tunneling dark currents in GeSn PDs. The presence of defect states inside the Ge_1–x_Sn_x_ active layer introduces the TAT of the carriers that is the source of the TAT-related-dark current, which depends on the tunneling the effective mass, defect states’ energy, and electric field in the depletion region [58]. Because of the close proximity of Γ-CB and L-CB in energy, electrons residing in both Γ-CB and L-CB participate in TAT to contribute to the dark current. According to the Hurkx model, the TAT-related dark current can be evaluated using the following calculation [59]:

(24) $$J_{\mathrm{TAT}}=J_{\mathrm{TAT}}^{\Gamma }+J_{\mathrm{TAT}}^{L}+J_{\mathrm{TAT}}^{h}=q\left[A_{e}^{\Gamma }G_{e}^{\Gamma }n_{i}^{\Gamma }+A_{e}^{L}G_{e}^{L}n_{i}^{L}+A_{h}G_{h}n_{i}\right]\times t_{i}$$

where $G_{e}^{\Gamma }$, $G_{e}^{L}$, and $G_{h}$ are the corresponding TAT enhancement factors, which can be calculated as [59]

(25) $$G_{e(h)}=\frac{\Delta E_{e(h)}}{k_{B}T}\int_{0}^{1}\exp\left[\frac{\Delta E_{e(h)}}{k_{B}T}u-K_{e(h)}u^{3/2}\right]du$$

(26) $$K_{e(h)}=\frac{4}{3}\frac{\sqrt{2m_{ce(h)}^{*}\Delta E_{e(h)}^{3}}}{q\hbar \left|F\right|}$$

where $\Delta E_{e(h)}$ is the energy difference between the defect state and CB minima (VB maxima), and F is the local electric field.

**Figure 16:**
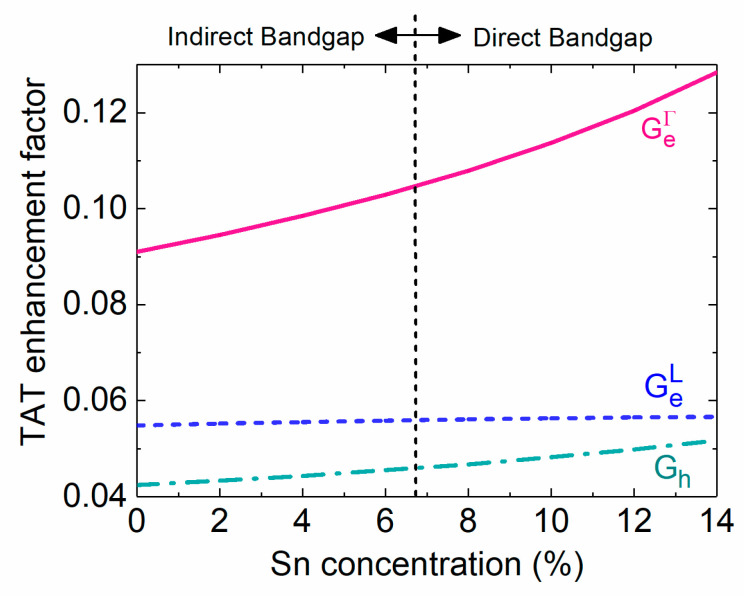
TAT enhancement factors as a function of the Sn concentration.

Figure 16 depicts the calculated TAT enhancement factors as a function of the Snconcentration. Because the zero-bias condition is considered in this analysis, TAT tunneling occurs due to the presence of the built-in electric field. The tunneling effective masses are reduced with the increase inthe Sn concentration, resulting in an increase in the TAT enhancement factors. Because Γ-CB electrons have a much smaller effective mass (0.045 − 0.166*x* + 0.043*x*^2^) than L-CB electrons (0.566 − 0.449*x* + 1.401*x*^2^ [47]), they are more likely to be captured by defect states, yielding higher TAT enhancement factors. On the other hand, the holes in the VB have a large effective mass, leading to low TAT enhancement factors.

**Figure 17:**
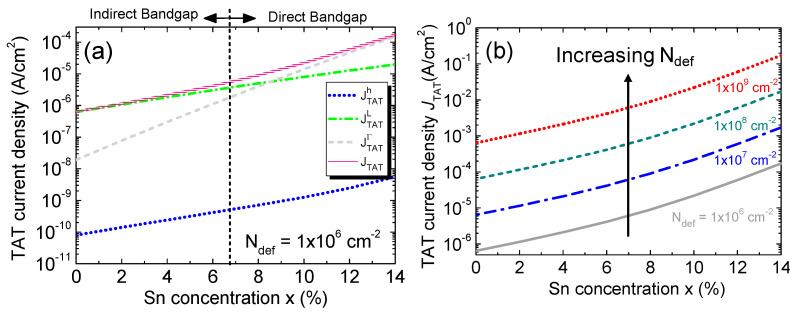
(**a**) Different contributors and total TAT current density as a function of the Sn concentration with a defect density of 1 × 10^6^ cm^−2^. (**b**) Total TAT current density as a function of the Sn concentration for different defect densities.

Figure 17a shows the calculated TAT current density, with its contributing component’s calculated defect density of *N*_def_ = 1 × 10^6^ cm^−2^ against the Sn concentration. Due to their lower effective mass, the electrons can tunnel at faster rates through the defect states than the holes. For lower Sn concentrations, the Γ-CB sits above the L-CB, so most electrons reside in the L-CB. As a result, the L-CB component is therefore higher than the Γ-CB component. Furthermore, the TAT dark current density increases with the increasing Sn concentration because of the reduction in bandgap energy that lowers the tunneling barrier, thereby increasing the tunneling probability and current. For higher Sn concentrations where Ge_1–x_Sn_x_ becomes direct-bandgap materials and the Γ-CB sits lower than the L-CB, more and more electrons populate the Γ-CB. Together with the small effective mass of Γ-CB electrons, the Γ-CB tunneling current goes up. Figure 17b shows the total TAT dark current density as a function of the Sn concentration for defect densities ranging from 1 × 10^6^ to 1 × 10^9^ cm^−2^. The TAT dark current density increases rapidly with the increasing defect density that causes the tunneling rate to go up.

### 4.6. Total Dark Current

**Figure 18:**
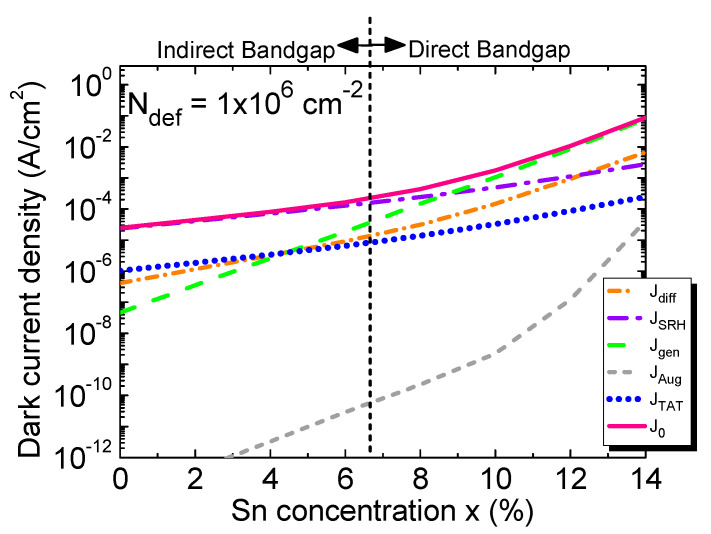
Total dark current density and its components as a function of the Sn concentration.

Following the above analysis of various dark current components, we are in the position to calculate the total dark current density. Figure 18 shows the calculated total dark current and its contributing components as a function of the Sn concentration at a fixed defect density of *N*_def_ = 1 × 10^6^ cm^−2^. In the case of pure Ge (x = 0%), the SRH and TAT components are the main contributors of the dark current, and this finding is in a good agreement with the reported result [58].The total dark current density, along with its contributing components, increases with the increasing Sn concentration. At lower Sn concentrations (x < 5%), SRH, TAT, and diffusion components dominate the dark current. At higher Sn concentrations, however, as Ge_1–x_Sn_x_ becomes a direct-bandgap material, the interband generation component takes over rapidly in regard to dominating the total dark current. Meanwhile, in the absence of an incident photon signal, the carrier concentration remains low in the *i*-Ge_1–x_Sn_x_ region, and the Auger contribution is therefore weak with respect to the other components throughout the Sn-concentration range of interest. Under the zero-bias condition, the electric field inside the intrinsic region is very small (~6 kV/cm), so the TAT current is therefore relatively insignificant in comparison with the SRH component.

**Figure 19:**
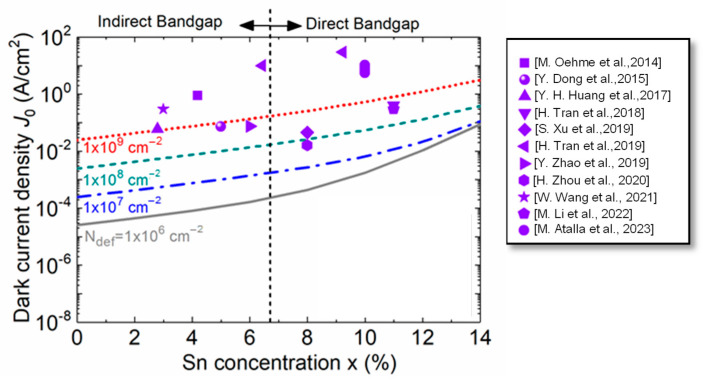
Calculated dark current density as a function of the Sn concentration for different defect densities (lines) compared with reported experimental results (scatters) at −1V bias voltage [31,32,33,34,35,36,37,38,39,40,42].

We next study the effect of higher defect densities on the dark current density. Figure 19 shows the calculated total dark current density as a function of the Sn concentration for defect densities ranging from 1 × 10^6^ to 1 × 10^9^ cm^−2^ and compares it with the previously reported experimental data [31,32,33,34,35,36,37,38,39,40,42]. For a fixed Sn concentration, the increase in the defect density increases the GR centers inside the *i*-Ge_1–x_Sn_x_ region, thereby increasing in the dark current. Because the dark current density is dominated by its SRH component at lower Sn concentrations (<8%), the total dark current increases sharply with the increase in the defect density. These results suggest that for Ge_1–x_Sn_x_ PDs with lower Sn concentrations, it is crucial to lower the defect density as much as possible in order to minimize the dark current. At higher Sn concentrations, however, as the *i*-Ge_1–x_Sn_x_ layer becomes a direct-bandgap material, the intrinsic carrier concentration increases rapidly with the bandgap reduction, and more of these carriers reside in Γ-CB, so the interband generation current becomes dominant. Thus, at higher Sn concentrations, the dark current density depends mostly on the material intrinsic properties and becomes less sensitive to the defect density. The analysis implies that a significant improvement in dark current is less likely to be obtained by further reducing the defect density to less than 10^7^ cm^−^². In addition, from the experimental data measured from the Ge_1–x_Sn_x_ PDs, it can be found that some experimental data fall in the range of the calculated dark current density between *N*_def_ = 1 × 10^6^ and 1 × 10^9^ cm^−2^, and some are higher than the calculated dark current density for *N*_def_ = 1 × 10^9^ cm^−2^. This discrepancy is attributed to the much thinner GeSn active layer of the reported GeSn PDs (typically 100–500 nm) which leads to much higher TAT currents. The results suggest that further optimization of GeSn PDs is necessary to minimize the dark current density of GeSn PDs.

## 5. Optical Responsivity Analysis

The optical responsivity is a figure of merit of the PDs which measures from the ratio of output electrical signal to input optical power. The optical responsivity of Ge_1–x_Sn_x_*p-i-n* PDs under normal incidence can be calculated as follows [29]:

(27) $$R_{\lambda }=\frac{q\lambda }{hc}\eta_{i}\left(1-R\right)\left[1-\exp\left(-\alpha t_{i}\right)\right]$$

where *λ* is the wavelength, *h* is the Planck’s constant, *η_i_* is the internal quantum efficiency, and *R* is the reflectivity of the anti-reflection coating on the top surface. To obtain the highest achievable responsivity of GeSn PDs, we assume that the internal quantum efficiency is 100% and that the reflectivity of the ARC layer is almost zero. (It should be noted that, in reality, the quantum efficiency of the GeSn PDs may be lower, and the reflectivity may not be zero, so the responsivity would be lower.) Another simplified assumption is made in our calculation that the absorption loss in the 100 nm thick *n*-Ge_1–x_Sn_x_ is neglected owing to the thin thickness. 

**Figure 20:**
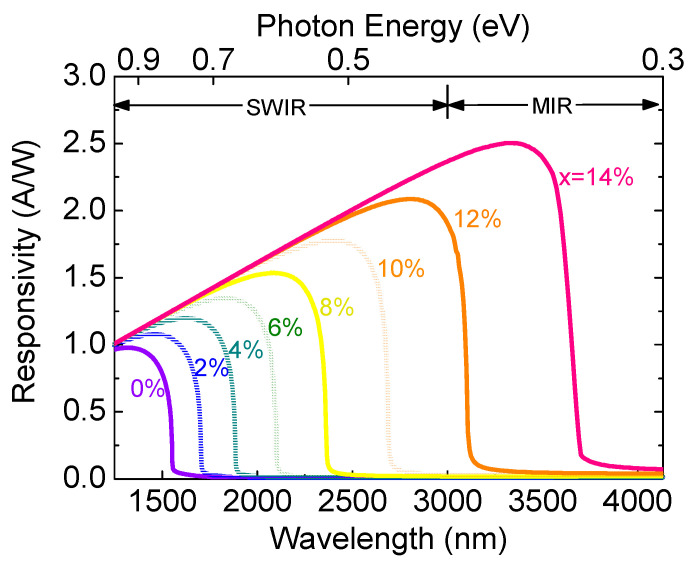
Optical responsivity spectra of GeSn PDs with different Sn concentrations.

Figure 20 shows the calculated optical responsivity spectra of the GeSn PD for different Sn concentrations. For a fixed Sn concentration, the optical responsivity increases with the increasing wavelength, and it sharply decreases at the direct-bandgap energy. As the Sn concentration increases, the cutoff wavelength redshifts owing to the reduced direct-bandgap energy. As a result, the optical responses of the GeSn PDs can cover the MIR region.

## 6. Detectivity Analysis

Finally, with the calculated total dark current density and optical responsivity, we then study one of the most important figures-of-merit for PDs, detectivity (D^*^). The detectivity of the Ge_1–x_Sn_x_PD can be expressed as follows [60]:

(28) $$D^{*}=\frac{R_{\lambda }\sqrt{A_{d}\Delta f}}{\sqrt{\langle i_{n}^{2}\rangle }}$$

where *A_d_* is the detection area, Δ*f* denotes the bandwidth and, *i_n_* is the dark current noise. The different contributors to dark current noise are thermal noise, shot noise, and generation–recombination noise. However, at room temperature, the magnitude of thermal noise is ~ten-to-one-hundred times more than the other contributing factors, suggesting that the effect of other contributorsis negligible with respect to the thermal noise [60]. Therefore, under dark conditions, $i_{n}^{}$ can be evaluated using the following calculation [19]:

(29) $$\langle i_{n}^{2}\rangle =\frac{4k_{B}T\Delta f}{R_{0}}$$

where *R*_0_ is the differential zero-bias resistance [53,60]. By combining Equations (28) and (29) with the dark current in Equation (5), the detectivity of the Ge_1–x_Sn_x_ *p-i-n* PD can be expressed as follows:

(30) $$D^{*}=\frac{R_{\lambda }}{2\sqrt{qJ_{0}}}$$

**Figure 21:**
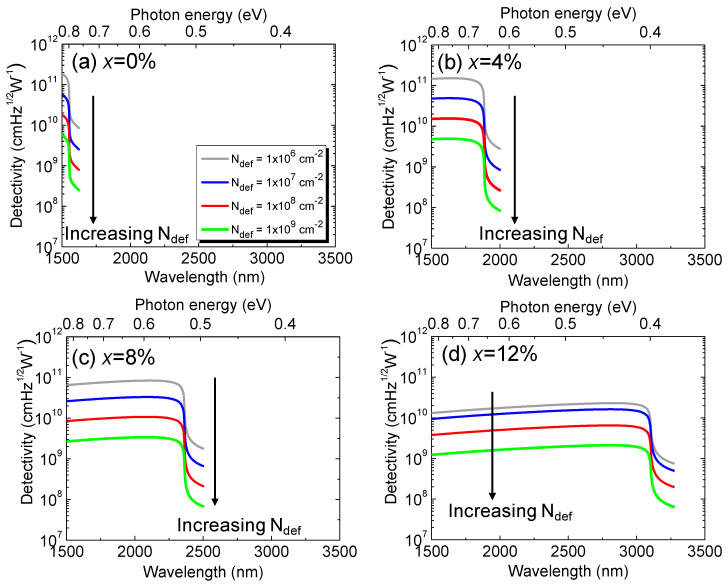
Calculated detectivity spectra of Ge_1–*x*_Sn*_x_* PDs for (**a**) x = 0%, (**b**) x = 4%, (**c**) x = 8%, and (**d**) x = 12% with various defect densities.

**Figure 22:**
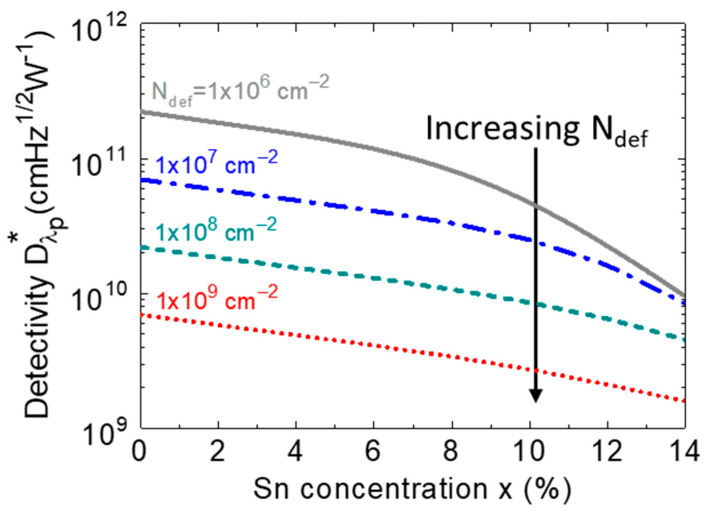
Variation in calculated detectivity as a function of the Sn concentration for different defect densities at a specified wavelength (λ_p_ = 0.9 × λ_c_).

Figure 21 shows the calculated detectivity of the Ge_1–x_Sn_x_ *p-i-n* PD under the zero-bias condition in the presence of various defect densities. For a fixed defect density and Sn concentration, the detectivity is higher at longer wavelengths, and then it rapidly decreases when reaching its cutoff wavelength. When the defect density increases, the detectivity drops, owing to the increased dark current density. From the calculated detectivity spectra, we extracted the peak detectivity (which is defined as the detectivity at a wavelength of *λ*_p_ = 0.9 × λ_c_) as a function of the Sn concentration, with various defect densities, and the results are depicted in Figure 22.The presence of higher defect states inside the intrinsic region acting as the GR centers produces a higher dark current. As a result, the detectivity decreases with the increasing defect density. Reducing the defect density can certainly improve detectivity. However, the degree to which the improvement can be obtained depends on the Sn concentration. It can be seen that, for smaller Sn concentrations (<8%), a significant improvement in detectivity can be achieved by reducing the defect density. However, when Ge_1–x_Sn_x_ becomes a direct-bandgap material at higher Sn concentrations, the payoff for detectivity by reducing the defect density to less than ~1 × 10^7^ cm^−2^ is not as impressive because the generation dark current becomes the dominant component. These results highlight the dependences of the defect density and Sn composition on the detectivity and photodetection range of GeSn PDs, providing useful guidelines for device and system developers to optimize GeSn PDs to achieve a desired performance for practical application.

## 7. Conclusions

In this paper, to illuminate practical applications, we theoretically studied the achievable performance of the homojunction Ge_1–x_Sn_x_ *p-i-n* PD at room temperature, owing to different sources of dark current, including minority carrier diffusion, various GR techniques, and tunneling of carriers. The presence of defect states produces GR centers that significantly impact the dark current. The calculation results show that the defect density significantly increases the dark current at low Sn concentrations, so improving the material quality is necessary and effective to suppress the dark current density and, thus, enhance the detectivity. On the other hand, when the Sn concentration is high enough to transfer the GeSn layers into a direct-bandgap material, the significantly reduced bandgap energy leads to a high intrinsic carrier density, so the generation dark current density dominates the dark current density. Thus, the detectivity is less sensitive to the defect density. Furthermore, the comparative study gives clear evidence that further optimization is needed to reduce the dark current. These results provide insights into the dark current density in terms of the defect density and Sn concentration of GeSn PDs, establishing important guidelines for device developers to improve the performance of GeSn PDs for practical applications.

## Figures and Tables

**Table 1:** Direct and indirect-band radiative recombination coefficients of Ge.

Radiative Recombination Coefficients (cm^3^ s^–1^)	Pure Ge [56]	This Work
Direct bandgap $\left(B_{eh}^{\Gamma }\right)$	1.3 × 10^–10^	1.75 × 10^–10^
Indirect bandgap $\left(B_{eh}^{L}\right)$	5.1 × 10^–15^	5.2 × 10^–15^

**Table 2:** Auger generation–recombination coefficients.

Auger Generation–Recombination Coefficients (cm^6^ s^–1^)	Pure Ge [56]	This Work
Γ-valley eeh $\left(C_{eeh}^{\Gamma }\right)$	–	2.62 × 10^–33^
L-valley eeh $\left(C_{eeh}^{L}\right)$ ehh $\left(C_{ehh}\right)$	3.0 × 10^–32^ 7.0 × 10^–32^	3.15 × 10^–32^ 7.31 × 10^–32^

## Data Availability

The data presented in this study are available upon request from the corresponding author. The data are not publicly available due to a commercial privacy policy.

## References

[1] Hoogeveen R.W.M., van der R.J., Goede A.P.H. (2001). Extended wavelength InGaAs infrared (1.0–2.4 μm) detector arrays on SCIAMACHY for space-based spectrometry of the Earth atmosphere. Infrared Phys. Technol..

[2] Arslan Y., Oguz F., Besikci C. (2015). Extended wavelength SWIR InGaAs focal plane array: Characteristics and limitations. Infrared Phys. Technol..

[3] Long M., Gao A., Wang P., Xia H., Ott C., Pan C., Fu Y., Liu E., Chen X., Lu W. (2017). Room temperature high-detectivity mid-infrared photodetectors based on black arsenic phosphorus. Sci. Adv..

[4] Deng G., Yang W., Gong X., Zhang Y. (2020). High-performance uncooled InAsSb-based pCBn mid-infrared photodetectors. Infrared Phys. Technol..

[5] Kimukin I., Biyikli N., Kartaloglu T., Aytur O., Ozbay E. (2004). High-Speed InSb Photodetectors on GaAs for Mid-IR Applications. IEEE J. Sel. Quantum Electron..

[6] Martin J.M., Hernandez J.L., Adell L., Rodriguez A., Lopez F. (1996). Arrays of thermally evaporated PbSe infrared photo-detectors deposited on Si substrates operating at room temperature. Semicond. Sci. Technol..

[7] Munoz A., Melendez J., Torquemada M.C., Rodrigo M.T., Cebrian J., de Castro A.J., Meneses J., Ugarte M., Lopez F., Vergara G. (1998). PbSe photodetector arrays for IR sensors. Thin Solid Films.

[8] Kasiyan V., Dashevsky Z., Minna Schwarz C., Shatkhin M., Flitsiyan E., Chernyak L., Khokhlov D. (2012). Infrared detectors based on semiconductor p-n junction of PbSe. J. Appl. Phys..

[9] Hu W.D., Chen X.S., Ye Z.H., Lu W. (2011). A hybrid surface passivation on HgCdTe long wave infrared detector with in-situ CdTe deposition and high-density hydrogen plasma modification. Appl. Phys. Lett..

[10] Hu W., Ye Z., Liao L., Chen H., Chen L., Ding R., He L., Chen X., Lu W. (2014). 128 × 128 long-wavelength/mid-wavelength two-color HgCdTe infrared focal plane array detector with ultralow spectral cross talk. Opt. Lett..

[11] Wang J., Xing Y., Wan F., Fu C., Xu C.H., Liang F.X., Luo L.B. (2022). Progress in ultraviolet photodetectors based on II-VI group compound semiconductors. J. Mater. Chem. C.

[12] Wang Y., Gu Y., Cui A., Li Q., He T., Zhang K., Wang Z., Li Z., Zhang Z., Wu P. (2022). Fast Uncooled Mid-Wavelength Infrared Photodetectors with Heterostructures of van der Waals on Epitaxial HgCdTe. Adv. Mater..

[13] Soref R. (2014). Silicon-based silicon-germanium-tin heterostructure photonics. Philos. Trans. R. Soc. Lond. Ser. A.

[14] Deen M.J., Basu P.K. (2012). Silicon Photonics: Fundamentals and Devices.

[15] Michel J., Liu J., Kimerling L.C. (2010). High-performance Ge-on-Si photodetectors. Nat. Photonics.

[16] Gupta J.P., Bhargava N., Kim S., Adam T., Kolodzey J. (2013). Infrared electroluminescence from GeSn heterojunction diodes grown by molecular beam epitaxy. Appl. Phys. Lett..

[17] Bauer M., Taraci J., Tolle J., Chizmeshya A.V.G., Zollner S., Smith D.J., Menendez J., Hu C., Kouvetakis J. (2002). Ge-Sn semiconductors for band-gap and lattice engineering. Appl. Phys. Lett..

[18] Chizmeshy A.V.G., Ritter C., Tolle J., Cook C., Menendez J., Kuovetakis J. (2006). Fundamental studies of P(GeH_3_)_3_, As(GeH_3_)_3_, and Sb(GeH_3_)_3_: Practical n-dopants for New Group IV Semiconductors. Chem. Mater..

[19] Chang G.-E., Yu S.-Q., Liu J., Cheng H.H., Soref R.A., Sun G. (2022). Achievable performance of uncooled homojunction GeSn mid-infrared photodetectors. IEEE J. Sel. Quantum Electron..

[20] Soref R. (2015). Mid-Infrared Photonics. Optical Fiber Communication Conference (OFC).

[21] Ghosh S., Bansal R., Sun G., Soref R.A., Cheng H.H., Chang G.E. (2022). Design and Optimization of GeSn Waveguide Photodetectors for 2-μm Band Silicon Photonics. Sensors.

[22] Mathews J., Roucka R., Xie J.Q., Yu S.Q., Menéndez J., Kouvetakis J. (2009). Extended performance GeSn/Si(100) p-i-n photodetectors for full spectral range telecommunication applications. Appl. Phys. Lett..

[23] Su S.J., Cheng B.W., Xue C.L., Wang W., Cao Q., Xue H.Y., Hu W.X., Zhang G.Z., Zuo Y.H., Wang Q.M. (2011). GeSn p-i-n photodetector for all telecommunication bands detection. Opt. Express.

[24] Roucka R., Mathews J., Weng C.G., Beeler R., Tolle J., Menéndez J., Kouvetakis J. (2011). High-Performance Near-IR Photodiodes: A Novel Chemistry-Based Approach to Ge and Ge–Sn Devices Integrated on Silicon. IEEE J. Quantum Electron..

[25] Xu S., Huang Y.C., Lee K.H., Wang W., Dong Y., Lei D., Panah S., Tan C.S., Gong X., Yeo Y.C. (2018). GeSn lateral p-i-n pho-todetector on insulating substrate. Opt. Express.

[26] Senaratne C.L., Wallace P.M., Gallagher J.D., Sims P.E., Kouvetakis J., Menéndez J. (2016). Direct gap Ge1-ySny alloys: Fabrication and design of mid-IR photodiodes. J. Appl. Phys..

[27] Gong X., Dong Y., Xu S.Q., Wang W. (2021). Germanium-tin (Ge_1–x_Sn_x_) photodetectors for 2 µm wavelength band. Jpn. J. Appl. Phys..

[28] Tran H., Pham T., Margetis J., Zhou Y., Dou W., Grant P.C., Grant J.M., Alkabi S., Du W., Sun G. Study of high performance GeSn photodetectors with cutoff wavelength up to 3.7 µm for low–cost infrared imaging. Proceedings of the CLEO: Science and Innovations 2019.

[29] Ghosh S., Kumar H., Mukhopadhyay B., Chang G.-E. (2021). Design and Modeling of High-Performance DBR-Based Resonant-Cavity-Enhanced GeSn Photodetector for Fiber-Optic Telecommunication Networks. IEEE Sens. J..

[30] McCarthy T.T., Ju Z., Schaefer S., Yu S.-Q., Zhang Y.-H. (2021). Momentum (k)-space carrier separation using SiGeSn alloys for photodetector applications. J. Appl. Phys..

[31] Oehme M., Kostecki K., Ye K.H., Bechler S., Ulbricht K., Schmid M., Kaschel M., Gollhofer M., Körner R., Zhang W.G. (2014). GeSn-on-Si normal incidence photodetectors with bandwidths more than 40 GHz. Opt. Express.

[32] Dong Y., Wang W., Lei D., Gong X., Zhou Q., Lee S.Y., Loke W.K., Yoon S.-F., Tok E.S., Liang G.C. (2015). Suppression of dark current in germanium-tin on silicon p-i-n photodiode by a silicon surface passivation technique. Opt. Express.

[33] Huang Y.-H., Chang G.-E., Li H., Cheng H.H. (2017). Sn-based waveguide p-i-n photodetector with strained GeSn/Ge multiple-quantum-well active layer. Opt. Lett..

[34] Tran H., Pham T., Du W., Zhang Y. (2018). High performance Ge0.89Sn0.11 photodiodes for lowcost shortwave infrared imaging. J. Appl. Phys..

[35] Xu S., Wang W., Huang Y.C., Dong Y., Masudy-Panah S., Wang H., Gong X., Yeo Y.C. (2019). High-speed photo detection at two-micron-wavelength: Technology enablement by GeSn/Ge multiple-quantum-well photodiode on 300 mm Si substrate. Opt. Express.

[36] Tran H., Littlejohns C.G., Thomson D.J., Pham T., Ghetmiri A., Mosleh A., Margetis J., Tolle J., Mashanovich G.Z., Du W. (2019). Study of GeSn Mid-infrared Photodetectors for High Frequency Applications. Front. Mater..

[37] Zhao Y., Wang N., Yu K., Zhang X.M. (2019). High performance silicon-based GeSn p–i–n photodetectors for short-wave infrared application. Chin. Phys. B.

[38] Zhou H., Xu S., Wu S., Huang Y.C., Zhao P., Tong J., Son B., Guo X., Zhang D., Gong X. (2020). Photo detection and modulation from 1,550 to 2,000 nm realized by a GeSn/Ge multiple-quantum-well photodiode on a 300-mm Si substrate. Opt. Express.

[39] Wang N., Xue C.L., Wan F.S., Zhao Y., Xu G.Y., Liu Z., Zheng J., Zuo Y.H., Chne B.W., Wang Q.M. (2021). High-performance GeSn photodetector covering all telecommunication bands. IEEE Photon. J..

[40] Li M., Zheng J., Liu X., Zhu Y., Niu C., Pang Y., Liu Z., Zuo Y., Cheng B. (2022). Sn composition graded GeSn photodetectors on Si substrate with cutoff wavelength of 3.3 μm for mid-infrared Si photonics. Appl. Phys. Lett..

[41] Nawwar M.A., Ghazala M.S.A., El-Deen L.M.S., El-Shaer A., Anis B., Kashyout A.E.H.B. (2022). Toward white light random lasing emission based on strained nanopolygermaniumdoped with tin via metal-induced crystallization (MIC). Cryst. Growth Des..

[42] Atalla M.R.M., Assali S., Koelling S., Attiaoui A., Moutanabbir O. (2023). Dark current in monolithic extended-SWIR GeSn PIN photodetectors. Appl. Phys. Lett..

[43] Nawwar M.A., Ghazala M.S., El-Deen L.M.S., Anis B., El-Shaer A., Elseman A.M., Rashad M.M., Kashyout A.E.H.B. (2023). Controlling barrier height and spectral responsivity of p-i-n based GeSn photodetectors via arsenic incorporation. RSC Adv..

[44] Karthik R., Sathyakam P.U., Mallick P.S. (2011). Effect of dislocation scattering on electron mobility in GaN. Nat. Sci..

[45] Ghosh S., Leonhardt D., Han S.M. (2014). Effect of threading dislocation density and dielectric layer on temperature-dependent electrical characteristics of high-hole mobility metal semiconductor field effect transistors fabricated from wafer-scale epi-taxially grown p-type germanium on silicon substrates. J. Appl. Phys..

[46] Wang Z., Liu J., Wang W., Chen H., Liu Z., Yu Q., Zeng H., Sun L. (2013). Aqueous phase preparation of graphene with low defect density and adjustable layers. Chem. Commun..

[47] Song Z., Fan W., Tan C.S., Wang Q., Nam D., Sun G. (2020). Band structure of Ge1–xSnx alloy: A full-zone 30-band k·p model. New. J. Phys..

[48] Chen H., Verheyen P., De Heyn P., Lepage G., De Coster J., Balakrishnan S., Absil P., Roelkens G., Van Campenhout J. (2016). Dark current analysis in high–speed germanium p–i–n waveguide photodetectors. J. Appl. Phys..

[49] Chang G.-E., Chang S.-W., Chuang S.L. (2010). Strain-Balanced GezSn_1-z_-Si_x_Ge_y_Sn_1-x-y_ Multiple-Quantum-Well Lasers. IEEE J. Quantum Electron..

[50] Giovane L.M., Luan H.-C., Agarwal A.M., Kimerling L.C. (2001). Correlation between leakage current density and threading dislocation density in SiGe p-i-n diodes grown on relaxed graded buffer layers. Appl. Phys. Lett..

[51] Scajev P., Soriute V., Kreiza G., Malinauskas T., Stanionyte S., Onufrijevs P., Medvids A., Cheng H.-H. (2020). Temperature dependent carrier lifetime, diffusion coefficient, and diffusion length in Ge_0.95_Sn_0.05_ epilayer. J. Appl. Phys..

[52] Gupta S., Simoen E., Loo R., Shimura Y., Porret C., Gencarelli F., Paredis K., Bender H., Lauwaert J., Vrielinck H. (2018). Electrical properties of extended defects in strain relaxed GeSn. Appl. Phys. Lett..

[53] Chuang S.L. (2012). Physics of Photonic Devices.

[54] Schubert E.F. (2006). Light-Emitting Diodes.

[55] Grzybowski G., Roucka R., Mathews J., Jiang L., Beeler R.T., Kouvetakis J., Menendez J. (2011). Direct versus indirect optical recombination in Ge films grown on Si substrates. Phys. Rev. B.

[56] Liu J., Sun X., Pan D., Wang X., Kimerling L.C., Koch T.L., Michel J. (2007). Tensile-strained, n-type Ge as a gain medium for monolithic laser integration on Si. Opt. Express.

[57] Sun G., Soref R.A., Cheng H.-H. (2010). Design of an electrically pumped SiGeSn/GeSn/SiGeSn double-heterostructure mid-infrared laser. J. Appl. Phys..

[58] Son B., Lin Y., Lee K.H., Chen Q., Tan C.S. (2020). Dark current analysis of germanium-on-insulator vertical p-i-n photodetectors with varying threading dislocation density. J. Appl. Phys..

[59] Hurkx G.A.M., Klaassen D.B.M., Knuvers M.P.G. (1992). A New Recombination Model for Device Simulation Including Tunneling. IEEE Trans. Electron Dev..

[60] Ghosh S., Bhattacharyya A., Sen G., Mukhopadhyay B. (2021). Optimization of different structural parameters of GeSn/SiGeSn Quantum Well Infrared Photodetectors (QWIPs) for low dark current and high responsivity. J. Comp. Electron..

